# Integrated molecular characterization of chondrosarcoma reveals critical determinants of disease progression

**DOI:** 10.1038/s41467-019-12525-7

**Published:** 2019-10-11

**Authors:** Rémy Nicolle, Mira Ayadi, Anne Gomez-Brouchet, Lucile Armenoult, Guillaume Banneau, Nabila Elarouci, Matthias Tallegas, Anne-Valérie Decouvelaere, Sébastien Aubert, Françoise Rédini, Béatrice Marie, Corinne Labit-Bouvier, Nicolas Reina, Marie Karanian, Louis-Romée le Nail, Philippe Anract, François Gouin, Frédérique Larousserie, Aurélien de Reyniès, Gonzague de Pinieux

**Affiliations:** 1 Programme Cartes d’Identité des Tumeurs (CIT), Ligue Nationale Contre le Cancer, Paris, France; 2 Department of Pathology, CHU de Toulouse – Oncopole, Université de Toulouse, Toulouse, France; 3 Plateforme de Génétique Moléculaire des Cancers, CHRU de Tours, Tours, France; 4 Department of Biopathology, Centre Léon Bérard, Lyon, France; 5 University of Lyon, Université Claude Bernard Lyon 1, CNRS 5286, INSERM U1052, Cancer Research Centre of Lyon, Lyon, France; 6 Department of Pathology, CHU de Lille, Université de Lille, Lille, France; 7 UMR1238 INSERM Université de Nantes, Sarcomes osseux et remodelage des tissus calcifiés, Faculté de médecine, NANTES, France; 8 Department of Pathology, CHU de Nancy, Nancy, France; 9 Department of Pathology, CHU de Marseille, Aix Marseille Université, INSERM, MMG, Marseille, France; 10 Department of Orthopedic Surgery, Hôpital Pierre-Paul Riquet, CHU de Toulouse, Toulouse, France; 11 Department of Orthopedic Surgery, CHRU de Tours, Université de Tours, Tours, France; 12 Department of Orthopedic Surgery, Hôpital Cochin, Assistance Publique-Hôpitaux de Paris, Université Paris Descartes, Sorbonne Paris Cité, Paris, France; 13 Department of Surgery, Centre Léon Bérard, Lyon, France; 14 Department of Orthopaedic Surgery, CHU Nantes, Nantes, France; 15 Service de Pathologie, Hôpital Cochin, AP-HP, Université Paris Descartes, Paris, France; 16 Department of Pathology, CHRU de Tours, Université de Tours, Tours, France

**Keywords:** Cancer genomics, Bone cancer, Sarcoma

## Abstract

Chondrosarcomas are primary cancers of cartilaginous tissue with highly contrasting prognoses. These tumors are defined by recurrent mutations in the IDH genes and other genetic alterations including inactivation of *CDKN2A* and *COL2A1*; however, these have no clinical value. Here we use multi-omics molecular profiles from a series of cartilage tumors and find an mRNA classification that identifies two subtypes of chondrosarcomas defined by a balance in tumor differentiation and cell cycle activation. The microRNA classification reveals the importance of the loss of expression of the 14q32 locus in defining the level of malignancy. Finally, DNA methylation is associated with IDH mutations. We can use the multi-omics classifications to predict outcome. We propose an mRNA-only classifier to reproduce the integrated multi-omics classification, and its application to relapsed tumor samples shows the progressive nature of the classification. Thus, it may be possible to use mRNA-based signatures to detect patients with high-risk chondrosarcomas.

## Abstract (web summary)

Chondrosarcomas are heterogenous tumours of the bone cartilage and have highly variable prognoses. Here, the authors perform a multi-omics analysis, revealing molecular features that can stratify clinical outcomes.

## Introduction

Chondrosarcoma is a heterogeneous type of primary bone cartilage malignancies with highly contrasting clinical outcomes. Chondrosarcomas may be distinguished by the body-location of the bone from which they arise, which may have an impact on the surgical resectability. Cartilage tumors may also be classified according to their location related to the bone, with a majority of central chondrosarcoma arising from the medullar cavity (more than 85%) and the less common peripheral and periosteal chondrosarcomas arising from the surface of the bone. Regardless of location, all chondrosarcomas are classified by their histology in three grades from the well-differentiated lowly cellular exceptionally metastatic grade I chondrosarcoma to the poorly differentiated and highly cellular grade III chondrosarcoma with 70% of patients developing metastasis^1^. In the context of high-grade cartilaginous tumors, the exceptionally aggressive dedifferentiated chondrosarcoma subtype may also be discerned. Decisive for clinical and surgical course of treatment, histological grading is to date the best predictor of clinical behavior. Unfortunately, histological grading is subject to high inter observer variability with disagreement between pathologist observed in a majority of cases^2^. This important limitation of histological analysis to select the most appropriate clinical management uncovers an urgent need for molecular markers to more robustly predict clinical behavior.

Recent genomic analyses focusing only on DNA mutations identified frequent activating mutations of isocitrate dehydrogenase genes *IDH1* and *IDH2*^3^ as well as inactivating mutations in the Collagen Type II Alpha 1 Chain *COL2A1*^4^. While of major biological relevance, neither of these alterations could be used to predict the prognosis of chondrosarcomas.

In this study, a series of 102 cartilage tumors (Supplementary Table 1, Supplementary Data 1) is collected from eight clinical centers in France between 1997 and 2013. This series is mostly composed of chondrosarcomas (*n* = 91, 89.2%) of all grades including 16 dedifferentiated chondrosarcomas. This series is used to uncover the molecular diversity of chondrosarcomas through the profiling of mRNA, microRNA, DNA methylation, DNA copy number aberrations, and DNA somatic mutations among those identified in previous whole-exome screening^4^.

A consensus clustering approach is applied separately to mRNA, microRNA, and DNA methylation profiles to classify chondrosarcomas into molecular subtypes. A multi-omics classification is then obtained by integrating the three molecular subtyping systems. This reveals three major molecular features delineating the diversity of clinical outcomes in chondrosarcomas: a high mitotic state, regional 14q32 loss of expression and *IDH* mutations leading to genome-wide DNA hypermethylation. These three robust and simple molecular features classify chondrosarcoma in subtypes with superior clinical value as compared to the current grading system.

## Results

### mRNA identifies a differentiation-proliferation balance

In order to classify chondrosarcomas based on the gene expression of tumor cells, the set of 102 mRNA transcriptomic profiles were first subject to an Independent Component Analysis (ICA), a blind source separation algorithm, to decompose the global transcriptomic components of chondrosarcoma. This revealed six components (Supplementary Fig. 1), two non-neoplastic components related to muscle tissue and hematopoietic lineages, three tumor-related components associated to the tumor’s differentiation, proliferation and glycolytic state, and one component associated to a technical metric. The gene expression measurements associated to non-neoplastic or technical components were removed prior to a class-discovery procedure. The unsupervised classification was obtained using an unsupervised consensus clustering approach, consisting in applying a hierarchical clustering method in a resampling framework to obtain a robust classification from high-dimensional datasets.

**Fig. 1:**
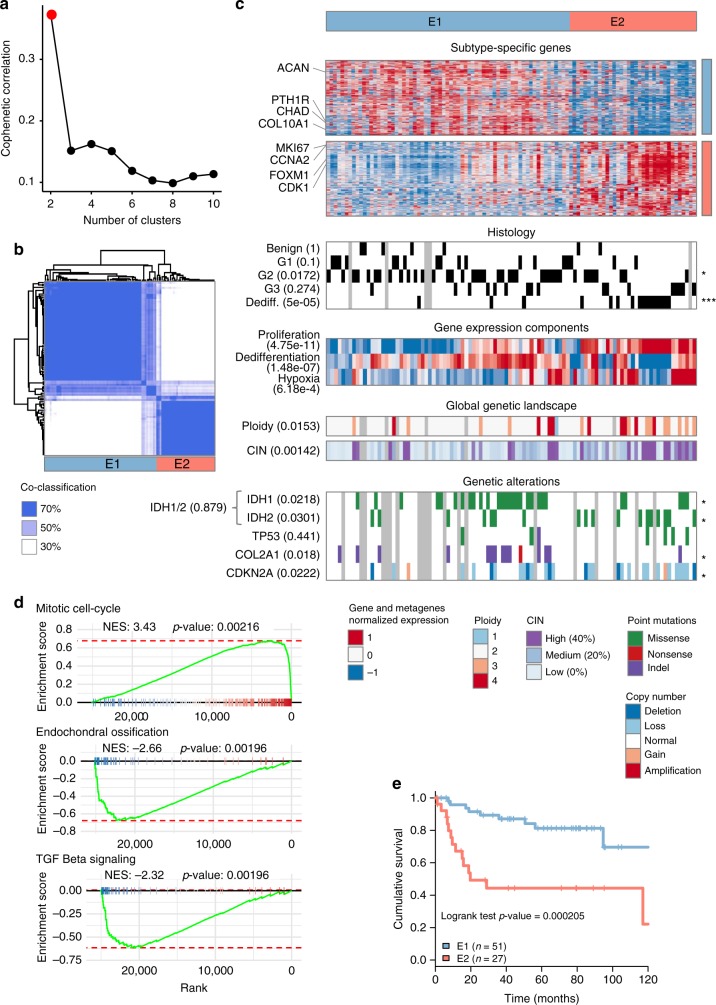
mRNA expression classification. **a** Cophenetic correlation coefficient for increasing number of clusters. Red point shows the chosen number of clusters (two) selected as the number previous to the largest decrease in the cophenetic correlation coefficient. **b** Heatmap of the co-classification matrix resulting from the consensus clustering approach. The matrix is symmetric and shows the frequency of co-classification of all 102 by 102 samples in the 1000 resampling iterations of the consensus clustering procedure at the selected cut of 2 clusters. **c** Characterization of the identified mRNA-based transcriptomic classification of chondrosarcoma using: subtype-specific marker gene expression, grading and histology features, metagenes extracted from the transcriptome profiles, general characteristic of genetic stability and gene-specific genetic alterations. When relevant, the association between the features shown as a heatmap and the two-class transcriptomic classification is shown (Student’s *t*-test for continuous variables or chi-squared test for discrete variables Significance of an FDR correction of the p-values are shown on the right of each line using the following encoding: ***FDR < 0.1%, **FDR < 1%, *FDR < 5%, and no symbol for FDR > 5%. **d** Gene Set Enrichment Analysis (GSEA) comparing E2 versus E1 samples. **e** Overall survival comparison of E1 and E2 tumors. Source data are provided as a Source Data file. CIN: Chromosomal instability index. Dediff Dedifferentiated chondrosarcoma, NES Normalized enrichment score

This revealed two robust mRNA-based subtypes (Fig. 1a, b). The first subtype E1 (*n* = 67) was defined by the over-expression of chondrogenic differentiation markers such as aggrecan, chondroadherin, or parathyroid hormone 1 receptor (*PTH1R*). The second subtype E2 (*n* = 35) lacked the expression of cartilage markers genes and was associated with high levels of cell-cycle related genes. Consistently, gene expression components levels, as well as pathway analysis (Fig. 1c, d) showed an overall increased in proliferation-related genes in the E2 subtype and higher levels of chondrogenic differentiation pathways, including of the TGF-beta signaling pathway, in E1. The association with histology was overall significant with a higher proportion of lower grade G1 and G2 in E1 while E2 was enriched in G3 and dedifferentiated chondrosarcomas (Fig. 1c). The analysis of genetic characteristics showed that tumors of the E2 proliferative subtype showed a higher chromosomal instability index and were more often polyploid. Furthermore, the deletion of the *CDKN2A* locus was prevalent in E2. Patient classified in the E2 subtype were associated to a poor overall survival with a hazard ratio (HR) of 4.29 (95% confidence interval [CI] 1.84–10; 36 months median overall survival E1: 86.77% [77.44%, 97.23%] versus E2: 44.32% [27.92%, 70.35%]) altogether highlighting the greater aggressivity (Fig. 1e and Supplementary Fig. 1).

### microRNA highlight the loss of expression of the 14q32 locus

**Fig. 2:**
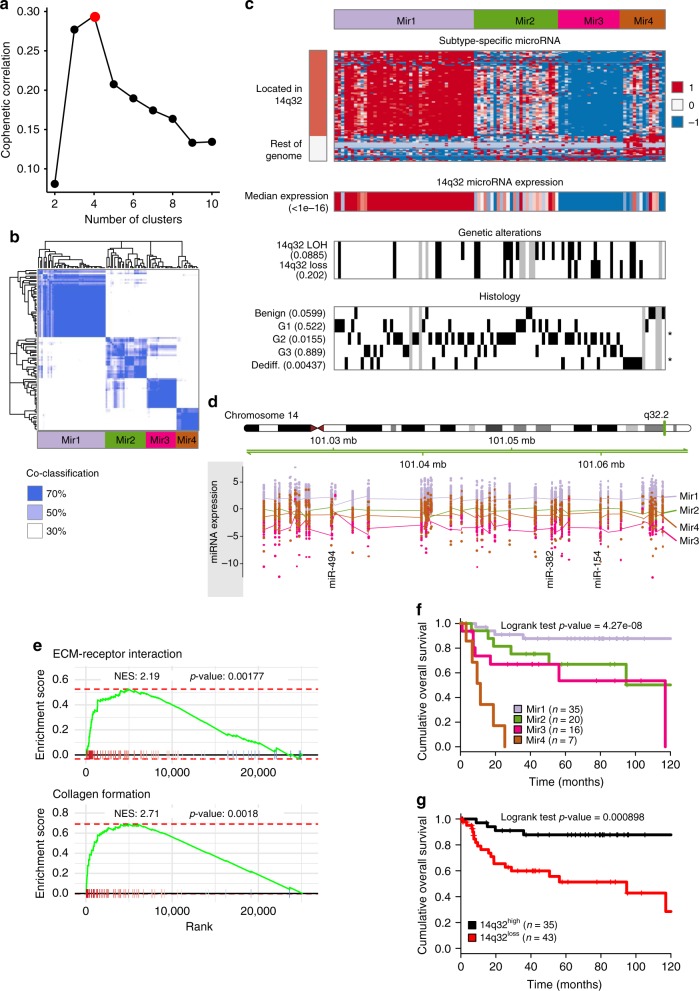
microRNA classification. **a** Cophenetic correlation coefficient for increasing number of clusters. Red point shows the chosen number of clusters (four) selected as the number previous to the largest decrease in the cophenetic correlation coefficient. **b** Heatmap of the co-classification matrix resulting from the consensus clustering approach. The matrix is symmetric and shows the frequency of co-classification of all 102 by 102 samples in the 1000 resampling iterations of the consensus clustering procedure at the selected cut of four clusters. **c** Characterization of the identified microRNA-based transcriptomic classification of chondrosarcoma using: subtype-specific microRNA expression annotated based on their genomic position, median expression of all microRNA in the 14q32 locus, genetic state of the 14q32 locus, and grading and histology. When relevant, the association between the features shown as a heatmap and the four-class micro-RNA classification is shown (Student’s *t*-test for continuous variables or chi-squared test for discrete variables). Significance of an FDR correction of the p-values are shown on the right of each line using the following encoding: ***FDR < 0.1%, **FDR < 1%, *FDR < 5%, and no symbol for FDR > 5%. **d** Sample-centered expression of microRNA plotted by their genomic position in the 14q32 locus. Each expression value is colored by the microRNA subtype and smoothened subtype-specific mean expression value with 95% confidence interval is shown. **e** Gene Set Enrichment Analysis (GSEA) comparing 14q32^high^ versus 14q32^low^ samples. **f** Overall survival comparison of the four microRNA subtypes. **g** Overall survival comparison of the 14q32^high^ samples, corresponding to the Mir1 subtype, versus the 14q32^low^ subtype comprising the Mir2, Mir3 and Mir4 subtypes. Source data are provided as a Source Data file. log-FC: log fold-change. LOH loss of heterozygosity, Dediff Dedifferentiated chondrosarcoma, NES Normalized enrichment score, ECM Extracellular matrix

Similarly to the analysis of the mRNA, the microRNA transcriptomes were first subject to an ICA decomposition (Supplementary Fig. 2), revealing two tumor-related components (proliferation and extracellular matrix interaction with loss of expression of the 14q32 locus) and one technically-related component (depth of microRNA sequencing). The consensus clustering of microRNA profiles after the removal of the microRNA associated to the technical component, revealed four robust microRNA-based subtypes (Fig. 2a, b) of which the most differential microRNAs were frequently found in the 14q32 locus (Fig. 2c). Three subtypes, Mir2 (*n* = 26), Mir3 (*n* = 19), and Mir4 (*n* = 14), were characterized by an overall decreasing level of expression of 86 microRNAs in this particular region of the genome (84% of the 102 total microRNAs measured in this region, FDR 1%). The Mir4 cluster was associated with an intermediate decrease in 14q32-located microRNA expression levels as well as to downregulation of other cancer-related microRNAs such as miR-27B, miR-125A, and miR-140. As the 14q32 locus is an imprinted region of the genome, we sought for genetic events that could explain such an effective regional silencing. Only few copy number losses or Loss of Heterozygosity (LOH) were identified and could be held accountable for the 14q32 regional loss of expression. The limited number of genetic alterations associated with the 14q32 loss of expression suggests additional epigenetic events, possibly beyond DNA methylation as none of the 178 microRNA-associated CpG measured could explain this regional loss of expression. Figure 2d shows the expression of the 102 microRNAs identified and quantified along the 14q32, illustrating broad regional silencing in all groups compared to the Mir1 cluster and highlighting miR-154, miR-382 and miR-384 previously shown to inhibit tumor growth in bone sarcomas^5–7^. Inhibition of the microRNAs located in the 14q32 locus was previously found to regulate proliferation in prostate cancer and osteosarcoma^8,9^. A pathway analysis of the genes correlating to the median expression of the 14q32 microRNA cluster revealed that 14q32 loss of expression was associated to the downregulation of pathways involved in extracellular matrix interactions and composition suggesting a remodeling of the tumor cell environment (Fig. 2e and Supplementary Fig. 2). The microRNA classification demonstrated a prognostic value (log-rank test *p*-value: 6.54e-8, Fig. 2f) and in particular patients belonging the subtypes characterized by the loss of expression of the 14q32 locus (i.e., Mir2, Mir3, and Mir4) were associated to the poorest prognosis with a hazard ratio of 5.04 (95% CI 1.71–14.9, Fig. 2g).

### DNA methylation mirrors *IDH* mutations

Similarly to the analysis of RNA profiles, the DNA methylation were first subjected to an ICA decomposition (Supplementary Fig. 3), revealing two tumor-related components (proliferation and differentiation) and one non-neoplastic related component (hematopoietic lineage).

**Fig. 3:**
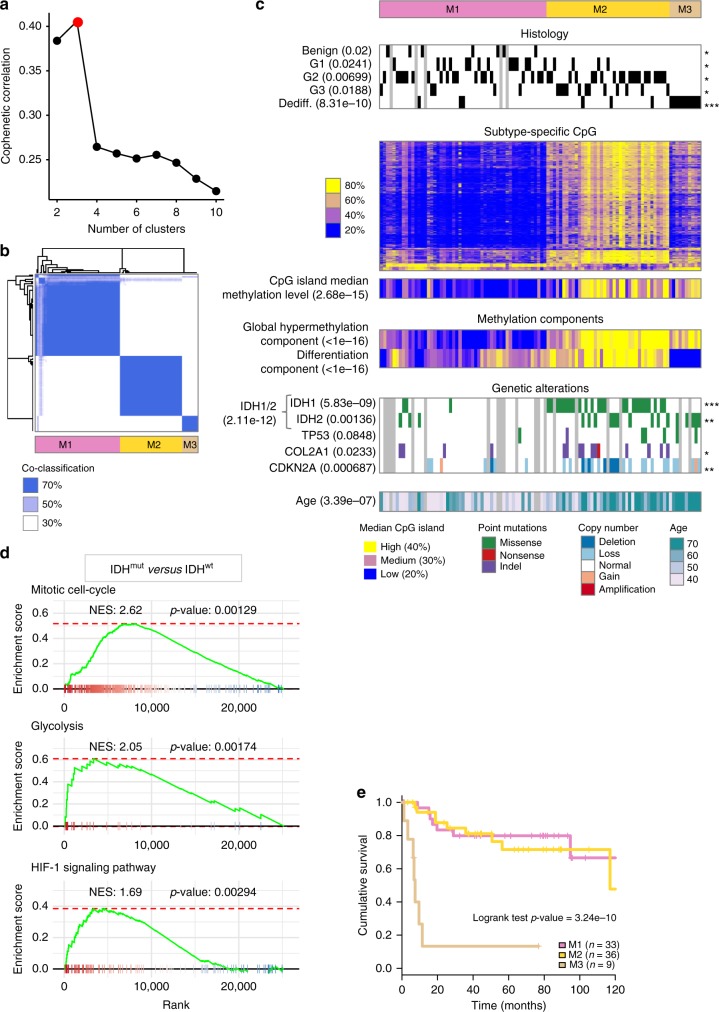
DNA methylation classification. **a** Cophenetic correlation coefficient for increasing number of clusters. Red point shows the chosen number of clusters (3) selected as the number previous to the largest decrease in the cophenetic correlation coefficient. **b** Heatmap of the co-classification matrix resulting from the consensus clustering approach. The matrix is symmetric and shows the frequency of co-classification of all 102 by 102 samples in the 1000 resampling iterations of the consensus clustering procedure at the selected cut of three clusters. **c** Characterization of the identified methylation-based classification of chondrosarcoma using: grading and histology features, subtype-specific CpG methylation level, median level of all CpG found in CpG islands, components extracted from the methylation profiles, gene-specific genetic alterations and age of the patient at diagnosis. When relevant, the association between the features shown as a heatmap and the three-class methylation classification is shown (Student’s *t*-test for continuous variables or Chi-squared test for discrete variables). Significance of an FDR correction of the *p*-values are shown on the right of each line using the following encoding: ***FDR < 0.1%, **FDR < 1%, *FDR < 5%, and no symbol for FDR > 5%. **d** Gene Set Enrichment Analysis (GSEA) comparing IDH^mut^ versus IDH^wt^ samples. (**e**) Overall survival comparison of M1, M2, and M3 tumors. Source data are provided as a Source Data file. Dediff Dedifferentiated chondrosarcoma, NES Normalized enrichment score

The consensus clustering of Chondrosarcoma DNA methylation profiles after the removal of the CpG associated to the non-neoplastic component, uncovered three subtypes (Fig. 3a, b): the *IDH*^wt^ subtype M1 (*n* = 53) covering all benign cartilage tumors and enriched in G1 chondrosarcomas, the *IDH*^mut^ M2 subtype (*n* = 39) enriched in high-grade G2 and G3 tumors, and a smaller third subtype M3 (*n* = 10) predominantly composed of dedifferentiated chondrosarcomas (Fig. 3c). *IDH*-mutated subtypes M2 and M3 displayed a hypermethylated genome as shown by the increase in the median level of CpG island methylation (Fig. 3c). *COL2A1* mutations and *CDKN2A* deletions were more frequently found in M2 tumors, which were also diagnosed in older patients than the M1 subtypes, suggesting that these tumors are more advanced. The M3 subtype is characterized by a low chondrogenic differentiation metagene, consistent with both the enrichment in the dedifferentiated histology as well as the downregulation of the endochondral ossification pathway (Fig. 3c and Supplementary Fig. 3). The *IDH* activating mutations driving a genome-wide hypermethylation in M2 tumors were found in both *IDH1* and *IDH2* genes. However, only *IDH2* activating mutations (R172S/W/T) were found in M3, suggesting differences in the effect of *IDH* mutations as previously shown by a higher production rate of 2-hydroxyglutarate in *IDH2* mutations^10^. Identification of the pathways upregulated in *IDH-*mutant samples shows that global hypermethylation in chondrosarcoma leads to the activation of proliferative and glycolytic state, the latter potentially driven by the hypoxia inducible factor^11^ (Fig. 3d). While several factors tend to indicate that *IDH*^mut^ subtypes M2 and M3 are more advanced tumors, no difference in survival was found between M2 and M1 tumors, indicating no prognostic impact of mutations in the *IDH* genes (Fig. 3e). However, patients in the M3 subtype were associated to a severe outcome with a median survival of 7.34 months and a hazard ratio of 13.7 (95% CI 4.51–41.3).

### Chondrosarcoma multi-omics classification

**Fig. 4:**
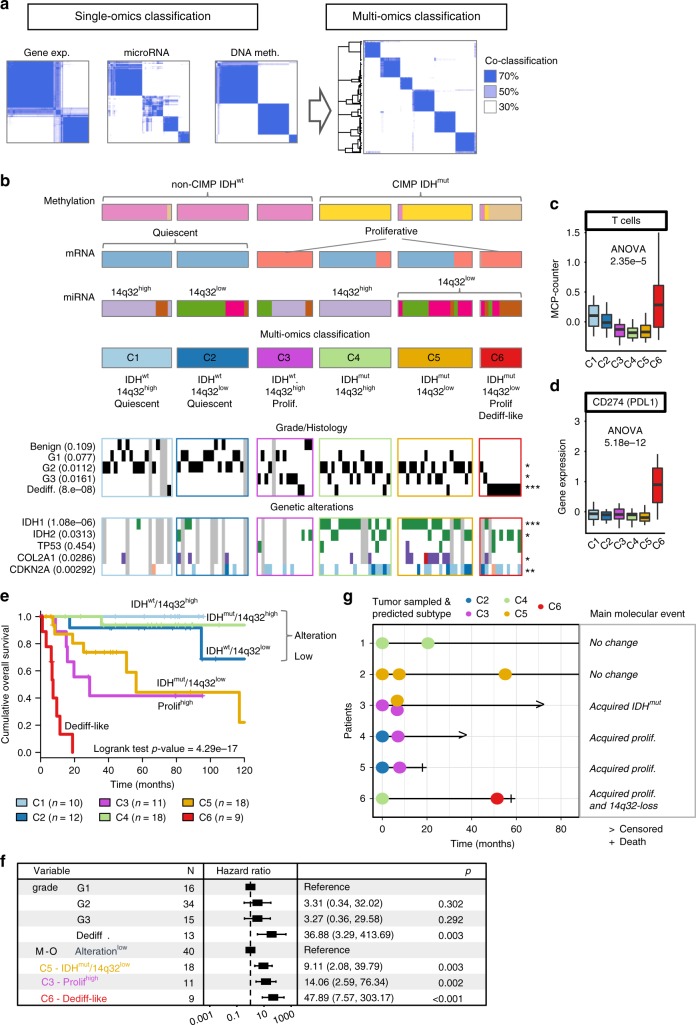
Multi-omics classification. **a** Schematic of a multi-omics classification of chondrosarcoma based on the three single-omics classifications. **b** Multi-omics classification of chondrosarcoma along each of the single-omics classification as well as its characterization using grading and histology features, and gene-specific genetic alterations. All of the 102 patient samples are represented as a column in the same order in each of the lines of the heatmaps. When relevant, the association between a feature shown as a heatmap and the 6-class multi-omics classification is shown (Student’s *t*-test for continuous variables or chi-squared test for discrete variables). Significance of an FDR correction of the p-values are shown on the right of each line using the following encoding: ***FDR < 0.1%, **FDR < 1%, *FDR < 5%, and no symbol for FDR > 5%. **c** Relative quantification of T lymphocytes cell population infiltration using MCP-counter and of **d** of the *PDL1* immune checkpoints. Boxplots show the median, the first and third quartile and whiskers extend to 1.5 times the interquartile range. **e** Overall survival comparison of the 6-class multi-omics classification. **f** Forest plot of the multi-variate analysis of survival including grade and the multi-omics classification after the simplification of the three alteration low subtypes into one (C1, C2, and C4). **g**. Follow-up study of patients with mRNA profiled relapse sample. *x*-axis shows time after initial diagnosis and each dot corresponds to a sample, including the initial sample. Dots are colored depending on the mRNA-based multi-omics subtype prediction. The right panel summarizes the main molecular events identified in at least one the relapse sample as compared to the initial sample, if any. Dediff Dedifferentiated chondrosarcoma

Single-omics classifications highlighted three major events in the carcinogenesis of chondrosarcomas: the acquisition of a proliferative state, the silencing of the 14q32 imprinted locus and the hypermethylation of DNA at a genome-wide level induced by *IDH* mutations. In order to unravel the combined effect of these molecular, a multi-omics classification was derived from the mRNA, microRNA, and methylation subtyping systems (Fig. 4a, b, Supplementary Fig. 4). In essence, the similarity between each pair of the 102 chondrosarcomas was computed as the mean of the probability of belonging to the same subtype in each single-omics classification system. A consensus clustering approach was then applied to this integrated multi-omics sample-similarity matrix in order to group samples by their similarity in all three molecular levels, potentially grouping single-omics subtypes into clusters with increased similarities when considering all molecular levels.

This revealed six subtypes that can be mostly distinguished by a combination of the three molecular characteristics: the *IDH* mutations and its hypermethylation effect (*IDH*^wt^ vs *IDH*^mut^), the 14q32 locus silencing (14q32^high^ vs 14q32^low^) and a low-differentiation/high-proliferation mRNA profile (Mitotic vs Quiescent). The two *IDH*^wt^/Quiescent subtypes (C1 and C2, *n* = 18 and *n* = 19, respectively) are enriched in G1 chondrosarcomas or in benign forms of cartilage tumors and are scarcely mutated in major genes. The *IDH*^wt^/14q32^high^/Mitotic subtype (C3, *n* = 15) is also rarely *IDH* mutated yet enriched in high-grade G3 tumors. The *IDH*^mut^/14q32^high^ subtype (C4, *n* = 19) is systematically *IDH* mutated similarly to the *IDH*^mut^/14q32^low^ subtype (C5, *n* = 20), which is also enriched in *CDKN2A* deletion and COL2A1 mutations. Finally, another IDH^mut^/14q32^low^ subtype was also identified and found to be enriched in the M3 methylation class, the Mir4 microRNA class, the E2 mRNA expression class as well as in the dedifferentiated histology tumors and was therefore termed dedifferentiated-like (C6 or dediff-like, *n* = 11). The analysis of the abundance of immune cell infiltration using MCPcounter^12^ revealed that the dedifferentiated-like subtype is enriched in T lymphocytes (Fig. 4c) and associated to a concomitant up-regulation of major immune checkpoint such as *PDL1* (Fig. 4d). These results suggest that this molecular definition of the most aggressive subtype of chondrosarcoma is defined by an immune-active microenvironment, as previously described for approximately half of the tumors histologically defined as dedifferentiated^13^.

The proposed multi-omics classification was highly associated with overall survival (Fig. 4e and Supplementary Fig. 4). In particular, the three subtypes that were defined as Quiescent and did not bear the combination of both *IDH*^mut^ and 14q32^low^ alterations (i.e., C1, C2, and C4), were associated to a highly favorable outcome and categorized as the alteration low group. This multi-omics classification remains relevant to predict survival outcome in a multi-variate model including the O’Neal et Ackerman grading system^14^ with substantial hazard ratios ranging from 9.05 (for the *IDH*^mut^/14q32^low^ C5 subtype, 95% CI 2.07–39.51) to 47.56 (95% CI 7.52–300.82) for the dedifferentiated-like C6 subtype (Fig. 4f). Given that the proposed multi-omics classification is based on three distinct type of molecular profiles, mRNA, microRNA, and DNA methylation, the possibility of using mRNA gene expression profiles alone to classify chondrosarcomas was investigated. Three gene expression only classifiers based on Support Vector Machine models were derived and tested in a cross-validation setting and were able to classify tumor samples with an accuracy of 96%, 95.1%, and 91.9% for the gene expression, DNA methylation and a simplified 14q32^high/low^ microRNA classification, respectively (Supplementary Fig. 4, Supplementary Data 2).

The multi-omics classification suggests a path describing chondrosarcoma progression in which *IDH* mutations and the silencing of the 14q32 locus are major steps driving the malignant progression. In order to investigate the progressive nature of the proposed classification, the transcriptomic profiles were generated from tumors that were resampled at relapse for a small group of patients. In total, eight relapsed tumor material was available for six distinct patients, among which four demonstrated a progression towards a more aggressive subtype including the acquisition of a mitotic phenotype, an *IDH*^mut^ profile or a loss of expression of the 14q32 locus (Fig. 4f). No mutations in the *IDH* genes were detected in the primary tumor associated to a relapse with an *IDH*^mut^ profile (patient 3 in Fig. 4f). This result substantiates a multistep carcinogenic process in chondrosarcoma in which the consecutive acquisition of a high-proliferation phenotype, the *IDH* mutation and the loss of expression of the 14q32 locus leads to a subtype of tumor with increasing aggressivity.

## Discussion

Altogether, the analysis of chondrosarcomas molecular profiles uncovered three major molecular features involved in cartilage tumor progression: *IDH* mutations implicating broad hypermethylation of the genome, the regional loss of expression of the 14q32 locus and in particular of the cluster of microRNAs at this location, and a transcriptomic state indicator of high mitotic potential. In addition, dedifferentiated chondrosarcomas, a histological entity with a dismal outcome, is defined by specific profiles in all three molecular dimensions analyzed here. The integration of these proposed single-omics features into a multi-omics classification revealed the effect of their combination on the aggressivity of cartilage tumors. This integrative molecular perspective of chondrosarcomas has superior prognostic value compared to the established grading system in this series and defines a subgroup representing approximately half of all chondrosarcomas as non-aggressive. It is also shown that a single mRNA-based profile is sufficient to obtain an accurate surrogate of the multi-omics classification. The analysis of relapsed samples suggests that chondrosarcoma tumors may progress to acquire aggressive features, potentially leading to adverse outcomes. It is still unclear at what point these features can be detected. Overall, our results support the importance of molecular diagnostic of chondrosarcomas for an accurate prognosis.

## Methods

### Patient series

Frozen tumor tissues were obtained in the context of diagnosis for 102 patients from different French hospitals (CHRU Tours, Hôpital Cochin APHP, CHU Toulouse, CHU Lille, Centre Léon Bérard, CHU Nancy, CHU Nantes and Hôpital la Timone APHM). The patient series was collected through the RESOS INCA network of bone with the support of the Groupe Sarcome Français - Groupe d’Etude des Tumeurs Osseuses (GSF – GETO). This retrospective study was approved by the French ethic committee in human research and agency in charge of non-interventional studies: Espace de reflexion ethique region centre (EREC: number of approval 2015 009; date of approval: February 16, 2015). With respect to regulatory procedures, the databases received authorizations from the Advisory Committee on Information Processing in Material Research in the Field of Health (CCTIRS) and the French Data Protection Authority (CNIL). The design of this retrospective study was done in agreement with the requirements for the use of biological material in research proposed by our institutional ethics guidelines. This study complies with all relevant ethical regulations for work with human participants, including concerning the systematic collection of informed consent.

### RNA isolation method

Two of ~50 mg fragments of frozen tissues were used per patient to obtain sufficient RNA yield. Tissues samples were transferred into homogenization tubes (Precellys lysing kit CK28R, Bertin Technologies) containing 1 mL TRIzol^®^ Reagent (Invitrogen™) with 200 µl Guanidine thiocyanate 4 M (Sigma–Aldrich™). Tissues were homogenized with the Precellys 24-Dual homogenizer (Bertin Technologies) three times 15 s at 6500 rpm speed with 10 s pause between homogenization steps. After grinding, a volume of lysis buffer (i.e., 1 mL Trizol reagent with 200 µl Guanidine thiocyanate 4 M) was added to each lysate bringing the total volume to 2400 μl. The phase separation step was carried out in two tubes by adding 200 μl of chloroform per 1200 μl of lysate. The aqueous phase was recovered after a 20 min centrifugation at 4 °C and 12000 × *g*, and one volume of 70% ethanol was added. Purification was then performed on a single purification column for each patient using the miRNeasy Mini kit (Qiagen™) according to the manufacturer’s instructions.

### DNA isolation method

DNA have been extracted from cartilage tumors tissues and cartilage non-tumors tissues from different French hospitals (CHRU Tours, Hôpital Cochin APHP, CHU Toulouse, CHU Lille, Centre Léon Bérard, CHU Nancy, CHU Nantes and Hôpital la Timone APHM). Approximately 50 mg fragments of frozen tissues were transferred into homogenization tubes (Precellys lysing kit CK28R, Bertin Technologies) containing 160 µl PBS (Sigma–Aldrich™). Tissues were homogenized with the Precellys 24-Dual homogenizer (Bertin Technologies) three times 15 s at 6500 rpm speed with 10 s pause between homogenization steps. A digestion step was then performed at 56 °C during 2 h in ATL buffer and proteinase K solution. Finally, DNA was purified using the QIAamp DNA Mini kit (Qiagen^TM^) according to the manufacturer’s instructions.

### Nucleic acids quality and samples selection

RNA and DNA concentrations were determined by the NanoDrop ND-1000 spectrophotometer at 260/280 nm (Nanodrop Technologies Inc). Quality and integrity of total RNA were analyzed with an Agilent 2100 Bioanalyzer (Agilent Technologies, UK) and DNA quality was checked with pre-cast 2% agarose gel (Invitrogen™). Results lead to the selection of 170 RNA samples and 177 DNA samples. A unique tumor sample from 102 patients had sufficient RNA and DNA quality and quantity to perform all omics analysis.

### Chondrosarcoma Copy Number Aberration and LOH using SNP arrays

Illumina OmniExpress v12 SNP chips were used to measure the genome-wide Copy Number profiles according to the manufacturer’s recommendations. Raw fluorescent signals were extracted using the BeadStudio software to obtain log R ratio (LRR) and B allele frequency (BAF) values. The tQN normalization procedure^15^ was used to correct the bias between the two dyes used in Illumina assays. The circular binary segmentation algorithm^16^ was used to segment genomic profiles and smooth log R ratio and B allele frequency values. The Genome Alteration Print (GAP) method was used to estimate ploidy, the level of non-tumor cell contamination and the allele-specific copy number of each segment^17^. Chromosomal instability index (CIN) was estimated by the mean number of SNP probes with a loss or gained status normalized by chromosomes length.

### Chondrosarcoma DNA CpG methylation profiling using microarrays

Illumina Infinium HumanMethylation450 chips were used to measure genome-wide CpG methylation profiles following manufacturer’s instructions. Beta-values were extracted using Illumina’s GenomeStudio software. Probes containing single-nucleotide and indel polymorphism or overlapping with a repetitive element that was not uniquely aligned to the human genome were removed.

### Chondrosarcoma microRNA profiling using RNAseq

Small RNA were selected from total RNA using the miRNeasy kit used to construct sequencing libraries based on previously published protocols^18^. Libraries were sequenced on an Illumina HiSeq system. Raw demultiplexed FASTQ files were quality controlled and further processed using the “Trim_adapter” script provided by the mirExpress software. sRNAbench^19^ software (version 10/14) was used to quantify read counts for each human microRNA referenced in mirBase21. Mature microRNA with more than two counts in more than two samples were kept for further analysis. miRNA counts were normalized using the upper-quartile method^20^.

### Chondrosarcoma mRNA transcriptomic profiling using microarray

The Ambion WT Expression Kit (Cat # 4411974) and the Affymetrix GeneChip^®^ WT Terminal Labeling Kit (Cat # 900671) were used to prepare biotinylated cDNA from 200 ng of total RNA. cDNA were hybridized to Affymetrix Human Gene 2.0 ST arrays. Chips were washed and strained in the GeneChip^®^ Fluidics Station 450 (Affymetrix) and scanned with the GeneChip^®^ Scanner 3000 7 G (Affymetrix) at a resolution of 0.7 µm. Raw.CEL data were extracted from the scanned images using the Affymetrix GeneChip^®^ Command Console (AGCC) version 4.0. CEL files were processed using the Affymetrix Expression Console software version 1.3.1. Raw probe set signal intensities were normalized using the Robust Multi-array Average (RMA) algorithm.

### Chondrosarcoma point mutations using a targeted gene panel

A custom gene panel involving all RefSeq exons of three genes (TP53, COL2A1 and CDKN2A) as well as the known IDH1 (V71 and R123) and IDH2 (R172) mutation hotspots. Targeted enrichment is performed with a PCR method on the Access Array microfluidic support from Fluidigm (as previously^21^). The genes IDH1, PTCH1, COL2A1, IDH2, TP53 were covered with 110 primer pairs designed with Primer3. The average size of the PCR is 291pb. Twelve control primers pairs are added for SNP genotyping and are used for sample-ID tracking. Forty-eight primers pools are created, each primer pair is present in three different pools. two hundred fifty nanogram of each purified sample are engaged on Access Array. This device allows the PCR combinations of 48 samples with 48 pools of primers. To process the 116 samples, a total of three Access Array are performed. The protocol of library preparation is following the description of the Access Array User Guide with Integragen optimizations (AmplIG) to increase the level of multiplexing. The output of one Access Array is 48 pools of 121 PCR. Each pool is subjected to a second round of PCR for eight cycles in a standard microplate format in order to add specific barcodes for sample identification and P5/P7 Illumina adapters for Illumina sequencing. The 48 pools recovered from the Access Array, corresponding to the specific enrichment of each sample, are controlled and quantified on Fragment Analyzer to perform 1 equimolar pool of 48 samples. This final pool is purified with SPRI beads and sequenced on a MiSeq V2 at 2 × 150b.

The bioinformatics analysis of sequencing data is based on the Illumina pipeline (CASAVA1.8.2). CASAVA performs alignment of a sequencing run to the reference sequence of each gene (hg19), calls the SNPs based on the allele calls and read depth, and detects variants (SNPs & Indels). The alignment algorithm used is ELANDv2 (performs multiseed and gapped alignments). Genetic variation annotation is realized from IntegraGen in-house pipeline. It consists on genes annotation (RefSeq), known polymorphisms (dbSNP 132, 1000Genome) followed by a mutation characterization (exonic, intronic, silent, and nonsense). For each position, the exomic frequencies (Homo & HTZ) are determined from all the exomes already sequenced at IntegraGen, and the exome results provided by HapMap, 1000Genome, EVS. Mutations were annotated using ANNOVAR^22^.

### Unsupervised classification of chondrosarcoma genome-wide profiles

Identical methodological approaches were applied to the three post-genetic genome-wide profiles, namely mRNA and microRNA transcriptomes and DNA CpG methylome. For each genome-wide profile dataset, a first analysis consisted in applying a dimension reduction technique, Independent Component Analysis, to identify the non-neoplastic and technical signals in each dataset. Then, a consensus clustering approach was applied independently to each dataset after the removal of non-neoplastic or technical components.

Independent Component Analysis (ICA) was used to extract biologically relevant components from transcriptome or methylation datasets. ICA was performed using the JADE (joint approximate diagonalization of eigenmatrices) algorithm^23^. The optimal number of independent components to select was determined by comparing the distance between samples in the original dataset and in the reduced dimensions. The optimal number of components was set as the one prior to the highest drop in distance-correlation gain, as shown in Supplementary Figs. 1 to 3. The sample projections and feature correlation to each component were then used to identify the source of each components.

Unsupervised classification was performed using a hierarchical consensus clustering approach. To remove the unwanted signal associated with non-neoplastic tissue contamination or technical bias, features with a high Pearson’s correlation with the unwanted components were removed (20%). The remaining probes were used to obtain a robust unsupervised classification by applying an extension of the ConsensusClusterPlus algorithm^24^. In brief, using a Pearson distance and Ward linkage, hierarchical clustering was resampled in 1000 iterations of resampling of a selection of the most variant features estimated by standard deviation (20% most variant for CpG methylation and mRNA, no selection for microRNA). For a given selection of k clusters (1 < k < 10), the result is a symmetric co-classification matrix with a number of rows and columns equal to the number of sample (*n* = 102) containing the frequency at which each pair of samples was found in the same cluster in the 1000 iterations. The consensus was given by a final hierarchical clustering using the complete linkage and the one minus the frequency of co-classification as sample distance. In order to select K, the number of clusters, the correlation coefficient between the final hierarchical clustering cophenetic distance and the original inter-sample distance was computed, resulting in the cophenetic correlation coefficient. The cophenetic distance between two samples is the height of the dendrogram at which the two branches that separately include both samples merge into a single branch. The optimal number of consensus cluster was selected as K preceding the largest drop in the cophenetic correlation coefficient.

### Chondrosarcoma mRNA classification

Among the 46,394 probes associated to autosome located genes, ICA was applied to the expression values of first half of probes with the highest standard deviation (total of 23,197 probes) following gene-wise zero-centering without variance scaling. Six independent components were retrieved using the last maximum inter-sample distance-correlation gain (Supplementary Fig. 1) Three components were considered to be associated with non-neoplastic tissue (ICA1 with muscle and ICA3 with hematopoietic cells) or technical bias (ICA6 associated to Affymetrix QC). The 9428 probes remaining after the removal of these component-associated genes were used for the unsupervised consensus classification task. Consensus clustering identified two mRNA clusters based on the cophenetic correlation coefficient.

### Chondrosarcoma microRNA classification

ICA was applied to the expression values of all of the 1566 microRNAs detected following mir-wise zero-centering without variance scaling. Three independent components were retrieved using the last maximum inter-sample distance-correlation gain (Supplementary Fig. 2, details in previous section classification methodology, subsection Independent Component Analysis). One component was considered to be associated with technical bias (ICA3). The 721 microRNAs remaining after the removal of the technical component-associated genes were used for the unsupervised consensus classification task (using 50% most variant microRNAs instead of 20%). Consensus clustering identified four microRNA clusters based on the cophenetic correlation coefficient.

### Chondrosarcoma DNA methylation classification

Among the 285,631 probes associated to autosome located CpG, ICA was applied to the methylation values of first half of probes with the highest Standard Deviation (total of 142,815 probes) following CpG-wise zero-centering without variance scaling. Three independent components were retrieved using the last maximum inter-sample distance-correlation gain (Supplementary Fig. 3, details in previous section classification methodology, subsection Independent Component Analysis). One component was considered to be associated with non-neoplastic tissue (ICA2 with hematopoietic cells). The 150,399 probes remaining after the removal of the non-neoplastic component-associated genes were used for the unsupervised consensus classification task. Consensus clustering identified three DNA methylation clusters based on the cophenetic correlation coefficient.

### Chondrosarcoma multi-omics classification

A multi-omics unsupervised classification was obtained by combining the results of the consensus clustering of each of the single-omics, namely mRNA, microRNA and DNA methylation. The sample co-classification matrix of each omics was averaged and the resulting combined matrix was used as an inter-sample multi-omics similarity matrix. Given the nature of the data used to construct the multi-omics classification (a similarity matrix instead of a data matrix), the consensus clustering for unsupervised classification approach used on the single-omics profiles was adapted. A Partition Around Medoids (PAM) approach was applied on the multi-omics similarity matrix in a resampled framework (1000 iterations) using the ConsensusClusterPlus implementation^24^. The optimal number of clusters was defined by computing the dispersion of the resulting consensus clustering matrix^25^. The dispersion coefficient estimates the level of agreement between all the resample run with a value ranging from 0 to 1 with 1 denoting a perfect consensus matrix for which all resample iteration provided the same output.

The resulting final multi-omics classification was composed of 6 classes as shown in Fig. 4 and Supplementary Fig. 4.

### Chondrosarcoma functional analysis

Pathway enrichment analysis was performed using a fast implementation of GSEA^26^. All GSEA were performed with a pre-ranked list of genes using either Pearson’s correlation to a given component’s sample projections or limma’s moderate t statistic when comparing two sets of samples. Pathways references included Reactome^27^ and KEGG^28^. WikiPathway was only used for its reference of the Endochondral ossification pathway. Immune and stromal cell infiltration were estimated using MCP-counter^12^. Associations between binary and discrete variables (e.g., mutations or grading and histology vs classification) were tested using the Chi-square test. Associations between discrete and continuous variables (e.g., histology vs independent components) were tested using ANOVA.

For each test, statistical significance was set at a two-sided *p-*value of <0.05.

### Survival analysis of patients with chondrosarcoma

Overall survival (OS) was defined as the time from surgery to death resulting from any cause. Relapse-free survival (RFS) was measured from the date of surgery to the time of relapse or death. All survival analyses were performed after the removal of benign samples including: three enchondromas, one chondroma, one osteochondroma, and one chondroblastoma-like. It is to be noted that all the survival analyses in this study were also done with the inclusion of benign samples and did not change any of the conclusions. Survival curves were estimated using the Kaplan–Meier technique and compared with the log-rank test. The Cox proportional hazard regression model was used for both univariate and multi-variate analyses and for estimating the hazard ratio with 95% confidence interval. Univariate and multi-variate Cox regression analyses as well as Kaplan–Meier curves were computed using the survival package of the R statistical suite. Forest plots were drawn using the forestmodel R package.

### Follow-up recurrence analysis and classification

Among the 102 patients included in the multi-omics analysis, six relapsed and were resampled. The mRNA of these recurrences, eight sample in total, were profiled using the same microarrays and processed in the same batch as the rest of the main series. In order to define the molecular class of these sample, a supervised classifier was devised using mRNA expression only for each of the omics classification: microRNA, mRNA, and DNA methylation.

For each omics, a Support Vector Machine (SVM) classifier with a linear kernel as implemented in the kernlab R package^29^ was used on the mRNA profiles of the 102 multi-omics series to predict the single-omics classifications. The two-class (E1 and E2) mRNA classifier was trained on a selection of the 200 most over-expressed genes (using a moderate t test) of each subtype. Similarly, the three-class (M1, M2, and M3) DNA methylation classifier was trained on a selection of the 200 most over-expressed genes (using a moderate *t*-test) of each subtype versus all others. The four-class microRNA classification was simplified to a two-class classification for this task: 14q32 loss of expression subtypes (Mir2, Mir3, and Mir4) were merged. All of the 93 differential genes (using a moderate t test, FDR = 5%) were used to train an SVM classifier. An evaluation of these three classifiers were performed in a 10-times repeated 10-fold cross-validation settings. Results of this evaluation for each single-omics classification is reported in Supplementary Fig. 4 showing average Accuracy measures from 91.9 to 96%. The 6-class multi-omics classification was defined using each of the single-omics classification mRNA-based classifier following the recursive partitioning diagram illustrated in Supplementary Fig. 4.

### Reporting summary

Further information on research design is available in the Nature Research Reporting Summary linked to this article.

## Supplementary information

Supplementary Information

Description of Additional Supplementary Files

Supplementary Data 1

Supplementary Data 2

Reporting Summary

Source Data

## Data Availability

The mRNA gene expression data, microRNA sequencing data, methylation data, and SNP array data are available from the ArrayExpress website under the accessions number E-MTAB-7264, E-MTAB-7265, E-MTAB-7263 and E-MTAB-8213 respectively. All the other data supporting the findings of this study are available within the article and its supplementary information files and from the corresponding author upon reasonable request.
