# Supplementary material for: Integrated molecular characterization of chondrosarcoma reveals critical determinants of disease progression
Source: Nat Commun. 2019 Oct 11;10:4622. doi: 10.1038/s41467-019-12525-7 (PMC6789144; doi:10.1038/s41467-019-12525-7)
Supplement: Supplementary file 1 — Supplementary Information [file 41467_2019_12525_MOESM1_ESM.pdf]

Supplementary information

Integrated molecular characterization of chondrosarcoma reveals critical determinants of disease progression

Nicolle et al.

			Number of patients (%)
**Gender**	Male		59 (57.8%)
	Female		39 (38.2%)
	Unknown		4 (3.92%)
**Tumor location**	Long bone		58 (56.9%)
	Flat bone		29 (28.4%)
	Elongated flat bone		6 (5.88%)
	Short bone		2 (1.96%)
	Irregular bone		2 (1.96%)
	Unknown		5 (4.9%)
**Histology**	Chondrosarcoma		91 (89.2%)
		G1*	18 (19.8%)
		G2*	39 (42.9%)
		G3*	17 (18.7%)
		Dedifferentiated*	16 (17.6%)
		Unknown*	1 (1.1%)
	Osteochondroma		2 (1.96%)
	Chondromas		5 (4.9%)
	Unknown		4 (3.92%)
			**Mean [range]**
**Age, in years**			53.69 [17, 88] (sd. 18.46)
	Unknown		7

*: percentage are shown among chondrosarcomas with n=90
sd. : Standard deviation

Supplementary Table 1
Series description.

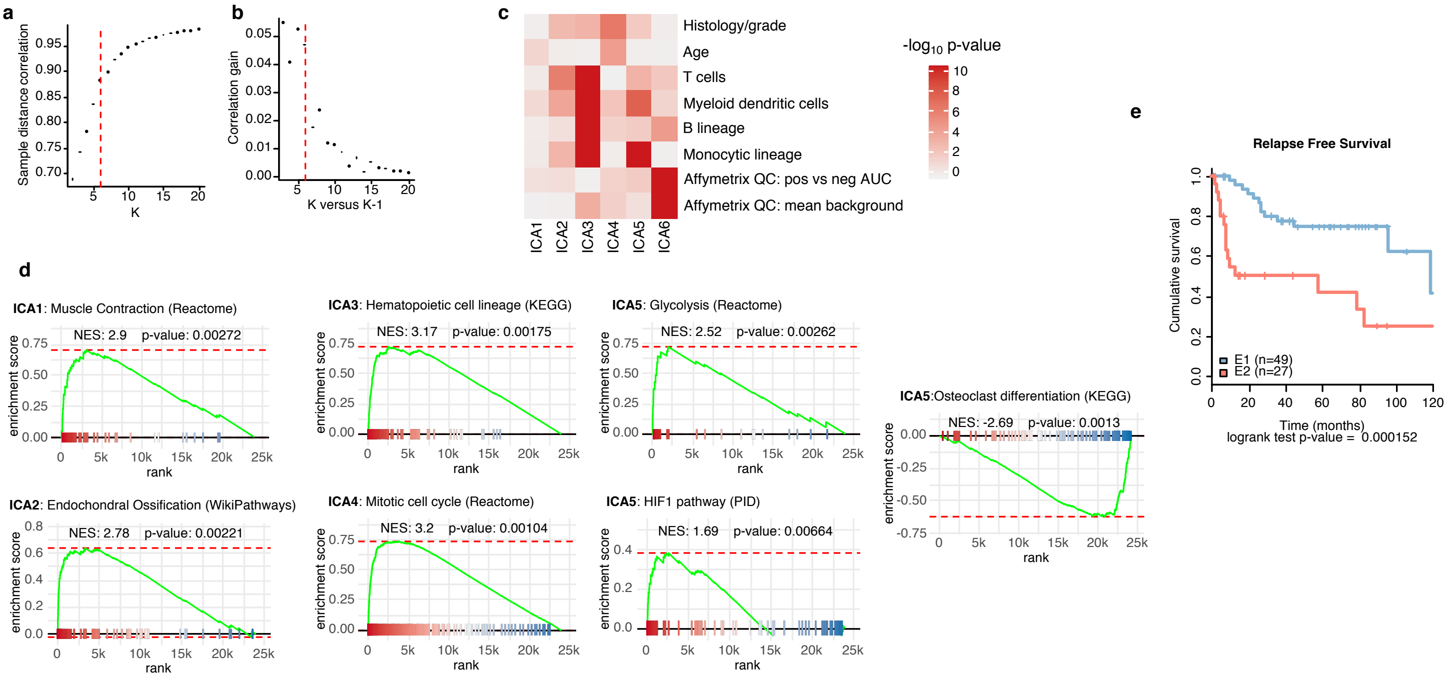

Supplementary figure 1

a. Inter-sample distance correlation between the original gene expression dataset and the Independent Component Analysis (ICA) reduced dimensions with increasing number of components. **b**. Differences, noted gain, between K and K-1 selected ICA components. Dashed red lines in a. and b. indicate the selected number of mRNA gene expression components, K=6, corresponding to a high gain in inter-sample distance correlation before the biggest drop in correlation gain. **c**. Heatmap representing the association, represented by the $-\log_{10}$ p-value, between each sample component and patient/sample characteristics. **d**. GSEA pathway enrichment of each ICA component. **e**. Comparison of the Relapse Free Survival between the two mRNA gene expression-defined subtypes E1 and E2.

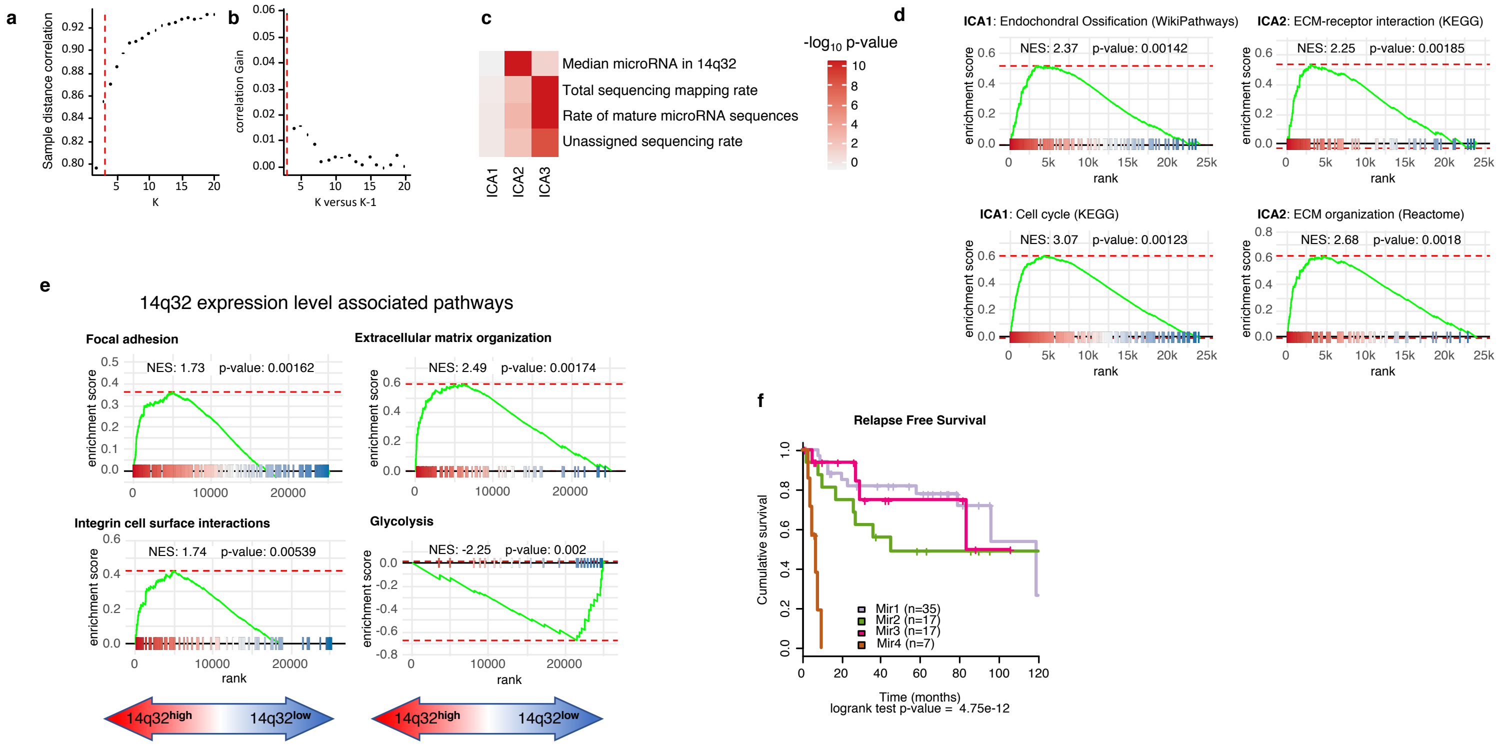

Supplementary figure 2

a. Inter-sample distance correlation between the original miRNA dataset and the Independent Component Analysis (ICA) reduced dimensions with increasing number of components. **b**. Differences, noted gain, between K and K-1 selected ICA components. Dashed red lines in a. and b. indicate the selected number of miRNA gene expression components, K=3, corresponding to a high gain in inter-sample distance correlation before the biggest drop in correlation gain. **c**. Heatmap representing the association, represented by the -$\log_{10}$ p-value, between each sample component and patient/sample characteristics. **d**. GSEA pathway enrichment of each non-technical ICA component (miRNA ICA3 is not shown as it was highly associated to the miRNA sequencing quality). **e**. GSEA pathways associated with the 14q32 locus miRNA expression level. **f**. Comparison of the Relapse Free Survival between the four miRNA gene expression-defined subtypes Mir1, Mir2, Mir3 and Mir4.

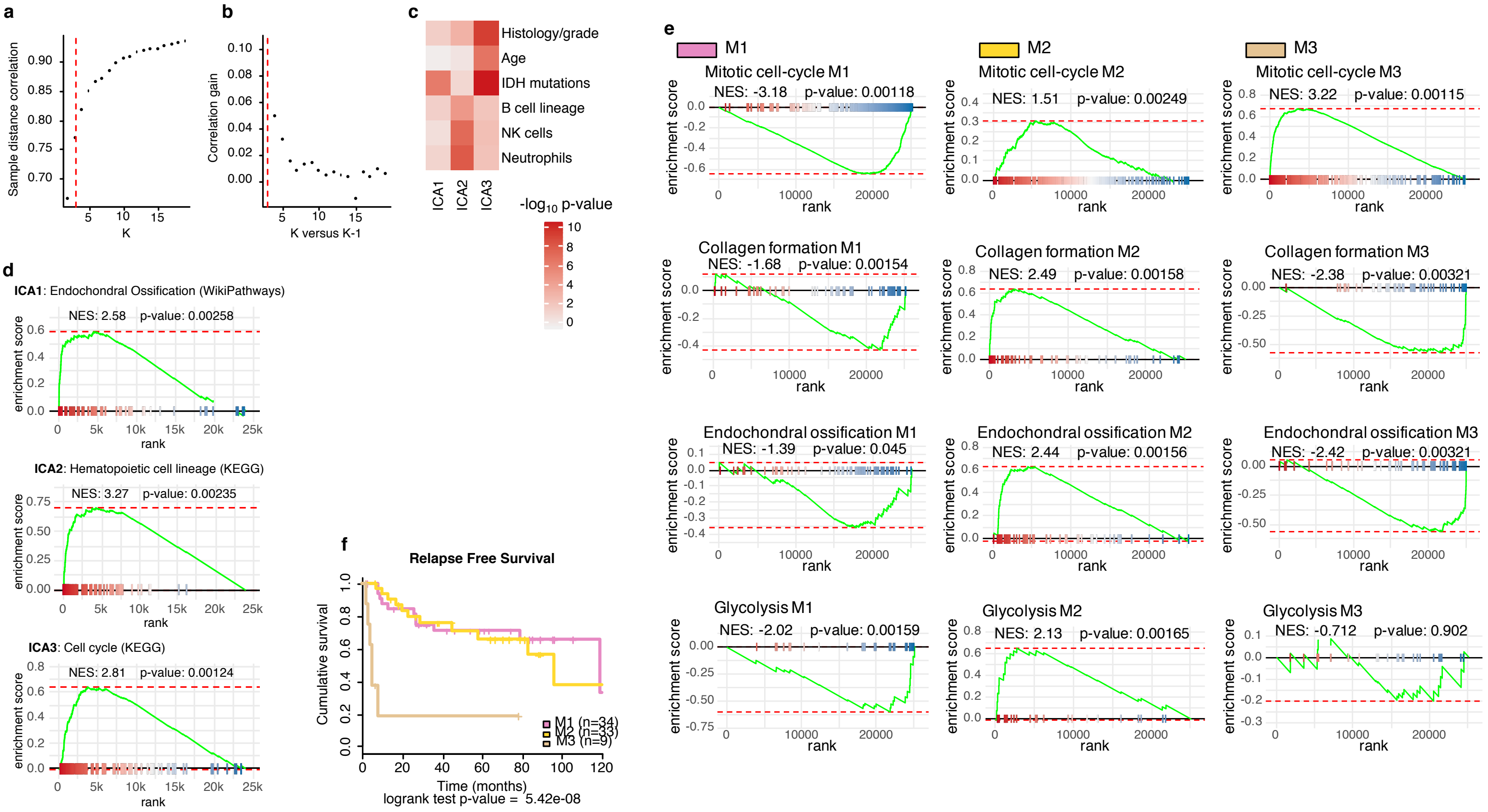

Supplementary figure 3

a. Inter-sample distance correlation between the original DNA methylation dataset and the Independent Component Analysis (ICA) reduced dimensions with increasing number of components. **b**. Differences, noted gain, between K and K-1 selected ICA components. Dashed red lines in a. and b. indicate the selected number of DNA methylation gene expression components, K=3, corresponding to a high gain in inter-sample distance correlation before the biggest drop in correlation gain. **c**. Heatmap representing the association, represented by the -log$_{10}$ p-value, between each sample component and patient/sample characteristics. **d**. GSEA pathway enrichment of each ICA component. **e**. GSEA pathways associated with each DNA methylation subtype. **f**. Comparison of the Relapse Free Survival between the three DNA methylation-defined subtypes M1, M2 and M3.

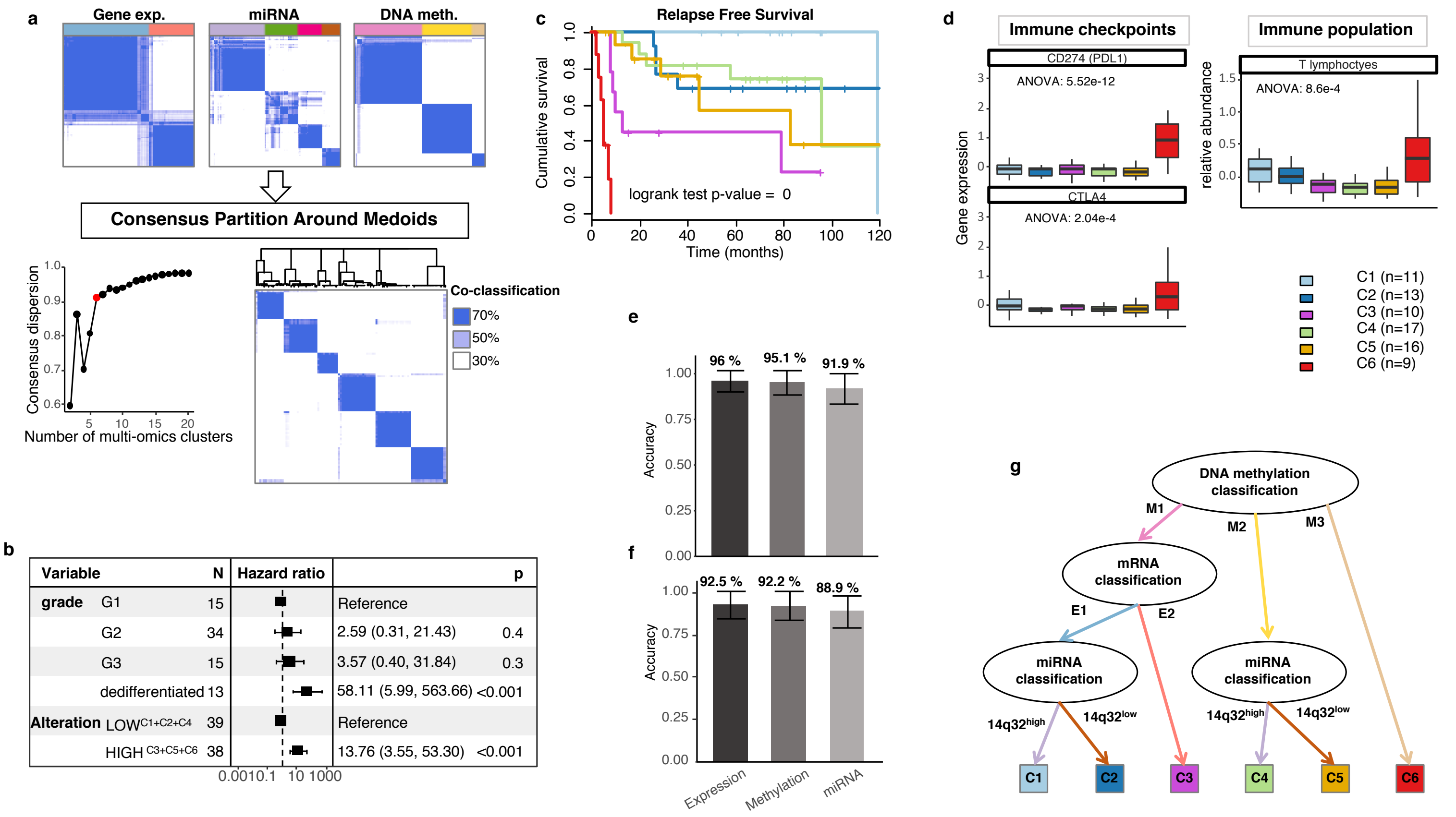

Supplementary figure 4

a. Multi-omics unsupervised classification based on single-omics consensus classifications **b**. Relapse Free Survival comparison of the 6-class multi-omics classification. **c**. Forest plot of the multivariate analysis of survival including grade and the multi-omics classification after the simplification into one alteration low (C1, C2 and C4) and one alteration high (C3, C5 and C6). **d.** Expression of immune genes and estimation of immune populations compared in the six multi-omics subtypes. **e.** and **f.** Average accuracy of single-omics subtype prediction using solely mRNA gene expression profiles. Average and standard deviation of a 10-times repeated 10-fold cross validation with prior (**e**) or cross-validated (**f**) gene selection. **g.** Recursive partitioning diagram used to define multi-omics subtype from single-omics classification.
