# Supplementary material for: Integrated molecular characterization of chondrosarcoma reveals critical determinants of disease progression
Source: Nat Commun. 2019 Oct 11;10:4622. doi: 10.1038/s41467-019-12525-7 (PMC6789144; doi:10.1038/s41467-019-12525-7)
Supplement: Supplementary file 2 — Description of Additional Supplementary Files [file 41467_2019_12525_MOESM2_ESM.pdf]

Description of Additional Supplementary Files

File Name: Supplementary Data 1
Description: Patient series description.

File Name: Supplementary Data 2
Description: mRNA-based classification signatures.
